# Hearing Loss Health Literacy Discrepancy Between Younger and Older Adults

**DOI:** 10.1093/geroni/igaa057.692

**Published:** 2020-12-16

**Authors:** Erika Squires, Hua Ou

**Affiliations:** Wayne State University, Detroit, Michigan, United States

## Abstract

Increasing the accessibility and affordability of hearing healthcare is a public health concern. Because low health literacy is a significant barrier to the use of existing effective healthcare services, it is critical to assess and understand health literacy deficits specific to hearing loss before implementing interventions. The purpose of this cross-sectional study was to identify differences in hearing loss health literacy among older and young adults, which is warranted because older adults are at-risk for lower levels of health literacy compared to their younger counterparts. Adults across the lifespan (n = 170) completed the Hearing Loss Health Literacy Assessment Tool, which includes self-rated ability to access/obtain, understand, and appraise hearing health information, as well as apply information to manage life with hearing loss. Results from an independent samples t-test indicated that older adults (M = 6.3, SD = 1.45, n = 54) self-reported significantly higher overall hearing health literacy than younger adults (M = 5.37, SD = 1.27, n = 116), t(168) = 4.22, p < 0.0001. Participants rated their ability to access/obtain information significantly lower than the other subscales. Age-differences in self-rated hearing health literacy exist. Findings from this study receive support from evidence indicating that the readability and suitability of the majority of patient education materials on hearing loss are not appropriate for the average U.S. adult. This investigation provides further evidence that the availability and accessibility of patient education materials on hearing loss is an important barrier that contributes to the limited use of hearing health care.

